# Tissue Distribution, Gender- and Genotype-Dependent Expression of Autophagy-Related Genes in Avian Species

**DOI:** 10.1371/journal.pone.0112449

**Published:** 2014-11-11

**Authors:** Alissa Piekarski, Stephanie Khaldi, Elizabeth Greene, Kentu Lassiter, James G. Mason, Nicholas Anthony, Walter Bottje, Sami Dridi

**Affiliations:** Center of Excellence for Poultry Science, University of Arkansas, Fayetteville, Arkansas, United States of America; University of Birmingham, United Kingdom

## Abstract

As a result of the genetic selection of broiler (meat-type breeders) chickens for enhanced growth rate and lower feed conversion ratio, it has become necessary to restrict feed intake. When broilers are fed *ad libitum,* they would become obese and suffer from several health-related problems. A vital adaptation to starvation is autophagy, a self-eating mechanism for recycling cellular constituents. The autophagy pathway has witnessed dramatic growth in the last few years and extensively studied in yeast and mammals however, there is a paucity of information in avian (non-mammalian) species. Here we characterized several genes involved in autophagosome initiation and elongation in Red Jungle fowl (*Gallus gallus*) and Japanese quail (*coturnix coturnix Japonica*). Both complexes are ubiquitously expressed in chicken and quail tissues (liver, leg and breast muscle, brain, gizzard, intestine, heart, lung, kidney, adipose tissue, ovary and testis). Alignment analysis showed high similarity (50.7 to 91.5%) between chicken autophagy-related genes and their mammalian orthologs. Phylogenetic analysis demonstrated that the evolutionary relationship between autophagy genes is consistent with the consensus view of vertebrate evolution. Interestingly, the expression of autophagy-related genes is tissue- and gender- dependent. Furthermore, using two experimental male quail lines divergently selected over 40 generations for low (resistant, R) or high (sensitive, S) stress response, we found that the expression of most studied genes are higher in R compared to S line. Together our results indicate that the autophagy pathway is a key molecular signature exhibited gender specific differences and likely plays an important role in response to stress in avian species.

## Introduction

Autophagy or cellular self-digestion, a lysosomal degradation pathway that is conserved from yeast to human, plays a key role in recycling cellular constituents, including damaged organelles [1]. Based on their mechanisms and functions, there are various types of autophagy, including micro- and macro-autophagy, as well as chaperone-mediated autophagy [2], [3]. The first two types have the capacity to engulf large structures through both selective (specific organelles such as mitochondria or endoplasmic reticulum referred to as mitophagy or reticulophagy, respectively [4], [5]) and non-selective mechanisms (bulk cytoplasm), whereas chaperone-mediated autophagy degrades only soluble proteins [6]. Micro-autophagy refers to the sequestration of cytosolic components directly by lysosomes through invaginations in their limiting membrane. However macro-autophagy refers to the sequestration within an autophagosome, a unique double-membrane cytosolic vesicle. Autophagosomes fuse with late endosomes and lysosomes, promoting the delivery of organelles, aggregated proteins and cytoplasm to the luminal acidic degradative milieu that enables their breakdown into constituent molecular building blocks that can be recycled by the cell [7].

A number of protein complexes (more than 30) and signaling pathways that regulate autophagy have been identified in yeast and many of these have mammalian orthologs (for review see [8]). These proteins can be grouped according to their functions at key stages of the autophagy pathway. The beclin-Vps34 complex is involved in the initiation of autophagosome formation. Beclin-1 enhances Vps34 activity [9] and binds to several partners that induce autophagy including ambra-1 [10], UVRAG [11], and bif-1 [12]. The second complex implicated in the initiation step of autophagosome formation is the ULK1/Atg1-Atg13-FIP200 complex [13]. Indeed, Atg13 binds ULK1 and when they are dephosphorylated they activate ULK1 that phosphorylates FIP200 to induce autophagosome formation [14]–[16]. For the autophagosome elongation, two ubiquitin-like systems are involved. The E1 ubiquitin activating enzyme-like, Atg7, activates Atg12 that is transferred to Atg10. Atg12 binds to Atg5 and then form a conjugate with Atg16L1 resulting in an 800-kDa complex [17] that is essential for the elongation of the pre-autophagosomal membrane. The second ubiquitin-like complex involves the protein microtubule-associated protein 1 light chain 3 (LC3/Atg8). LC3 is cleaved by Atg4B to form the cytosolic isoform LC3-I [18]. LC3-I is conjugated to phosphatidylethanolamine in a reaction involving Atg7 and Atg3 to form LC3-II which in turn targeted to elongating autophagosome membrane [19]. For the maturation and fusion stage, autophagosome moves, via dynein motor proteins [20], [21] towards the microtubule organizing center where the lysosomes are enriched. Autophagosome fuses with lysosome in a reaction involving several proteins including ESCRT [22], SNAREs [23], [24], Rab7 [25], [26], and the class C Vps proteins [27]. Recently, it has been reported that beclin-1 functions in the maturation of autophagosome through interaction with Rubicon [28].

Autophagy is essential for maintaining cellular homeostasis and autophagy malfunction is associated with diverse diseases such as neurodegeneration [29], cancer [30], immunity [31] and metabolic syndrome [32]. The amount of research focused on the autophagy pathway has witnessed dramatic growth in the last few years and the bulk of data are mainly originated from yeast and mammals. There is, however, a paucity of information on avian (non-mammalian) species. Therefore, the present study aimed firstly to characterize autophagy-related genes and their tissue distribution in chicken and quail, and secondly to determine their regulation by gender and genotype.

## Materials and Methods

### Ethic Statement

The present study was conducted in accordance with the recommendations in the guide for the care and use of laboratory animals of the National Institutes of Health and the protocol was approved by the University of Arkansas Animal Care and Use Committee under protocols 13039 and 10025.

### Animals

#### Chickens

Red Jungle fowl male and female chickens (*Gallus gallus*) (body weight average 1049±52 and 1654±67 g for female and male, respectively) were reared in floor pen under environmentally controlled facilities and under standard poultry rearing conditions (22±3°C for temperature and 50±5% relative humidity). Chickens were supplied with food (12.6 MJ·kg−1, 22% protein) and water available *ad libitum*.

#### Japanese quails

In order to assess whether the expression of autophagy-related genes is regulated by genotype, two lines of male Japanese quails (*coturnix coturnix Japonica*) were used. These two lines were established by long-term divergent selection for circulating corticosterone response to restraint stress, after which the low stress line (resistant, R) had 66% low plasma corticosterone levels compared to their high stress (sensitive, S) counterpart [33]. Quails of each genetic line were reared separately in floor pen under environmentally controlled facilities and were allowed *ad libitum* access to water and food (12.6 MJ·kg−1, 22% protein).

Animals were killed by cervical dislocation and tissues (liver, leg and breast muscle, brain, gizzard, intestine, heart, lung, kidney, adipose tissue, ovary and testis) were removed, immediately snap frozen in liquid nitrogen, and stored at −80°C until use.

### RNA isolation, reverse transcription and quantitative real-time PCR

Total RNA was extracted from chicken and quail tissues by Trizol reagent (catalog #15596018, Life Technologies) according to manufacturer’s recommendations, DNAse treated and reverse transcribed (catalog #95048-100, Quanta Biosciences). RNA integrity and quality was assessed using 1% agarose gel electrophoresis and RNA concentrations and purity were determined for each sample by Take 3 micro volume plate using Synergy HT multi-mode microplate reader (BioTek, Winooski, VT). The RT products (cDNAs) were amplified by real-time quantitative PCR (Applied Biosystems 7500 Real-Time PCR system) with Power SYBR green Master Mix (catalog #4312074, Life Technologies). Oligonucleotide primers used for avian autophagy-related genes are summarized in Table 1. The qPCR cycling conditions were 50°C for 2 min, 95°C for 10 min followed by 40 cycles of a two-step amplification program (95°C for 15 s and 58°C for 1 min). At the end of the amplification, melting curve analysis was applied using the dissociation protocol from the Sequence Detection system to exclude contamination with unspecific PCR products. The PCR products were also confirmed by agarose gel and showed only one specific band of the predicted size. For negative controls, no RT products were used as templates in the qPCR and verified by the absence of gel-detected bands. Relative expressions of target genes were determined by the 2^–ΔΔCt^ method [34].

**Table 1:** Oligonucleotide PCR primers.

Gene	Accession number^a^	Primer sequence (5′ → 3′)	Orientation	Product size (bp)
Beclin 1	NM_001006332	TGCATGCCCTTGCTAACAAA	Forward	61
		CCATACGGTACAAGACGGTATCTTT	Reverse	
Atg3	NM_001278070	GAACGTCATCAACACGGTGAA	Forward	65
		TGAGGACGGGAGTGAGGTACTC	Reverse	
Atg5	NM_001006409	TCACCCCTGAAGATGGAGAGA	Forward	66
		TTTCCAGCATTGGCTCAATTC	Reverse	
Atg9A	NM_001034821	AGTATGCCTCCACTGAGATGAGTCT	Forward	65
		GGCATGCTGCTTGTGCAA	Reverse	
Atg10	XM_424902	CATCTCACCAGATCTCAAGAAGGA	Forward	62
		CGACATGCGTAAGCAACGTT	Reverse	
Atg12	XM_003643073	GCACCCGCACCATCCA	Forward	61
		GAGGCCATCAGCTTCAGGAA	Reverse	
Atg14	XM_426476	GCGCTGCGAGGGTGTTAAT	Forward	61
		TTCTGTTACAAAAGCGTTCCTTGA	Reverse	
Atg13	XM_003641387	GGTCCCCCGAGCCAAATA	Forward	55
		ATGAGGTGCGGGAGCTGTAG	Reverse	
Atg7	NM_001030592	ACTGGCAATGCGTGTTTCAG	Forward	57
		CGATGAACCCAAAAGGTCAGA	Reverse	
Atg4B	NM_213573	CCCCGATGAAAGCTTCCA	Forward	56
		GCTCAGCGATGCTCATTCTG	Reverse	
Atg4A	NM_001271986	CACAGCAGTGCACATTTGCA	Forward	62
		CAGAGTCCTGCTGCGTTCCT	Reverse	
Atg16L1	XM_003641751	TGCATCCAGCCAAACCTTTC	Forward	57
		CGACGCTGGTGGCTTGTC	Reverse	
UVRAG	NM_001030839	GGGCTCATGGTCAGATGTGA	Forward	57
		CTTTGGAACGGGAATTGCA	Reverse	
Ambra 1	XM_001233288	GGGATGTTGTGCCTTTGCA	Forward	67
		CCTGGTGTGGGAAGAGAGAAGA	Reverse	
18S	AF173612	TCCCCTCCCGTTACTTGGAT	Forward	60
		GCGCTCGTCGGCATGTA	Reverse	

a Accession number refer to Genbank (NCBI).

### Multiple alignement and molecular evolution

Sequence alignments and percentage of amino acid conservation were assessed with the Clustal W and MUSCLE multiple alignment algorithms [35], [36] using chicken (non-mammalian) and mammalian autophagy-related gene (beclin1, Atg3, Atg5, Atg9a, Atg10, Atg12, Atg14, Atg13, Atg7, Atg4b, Atg4a, Atg16L1, UVRAG, Ambra1) sequences from database (see Table 2 for GenBank accession numbers). The phylogenetic tree based on these nucleotide sequence alignments was constructed using the neighbor-joining method of the MEGA6 program [37].

**Table 2:** Multiple alignment of the amino acid sequences of chicken autophagy-related genes with their mammalian orthologs.

	SPECIES					
GENE	Human	Mouse	Rat	Horse	Pig	Bovine
**Chicken Beclin 1**	75.97	73.87	74.03	75.26	72.28	75.36
(NM_001006332)	(NM_003766)	(NM_019584)	(NM_001034117)	(XM_005597370)	(XM_005668792)	(NM_001033627)
**Chicken Atg3**	81.84	81.6	79.7	75.57	80.33	91.53
(NM_001278070)	(NM_001278712)	(NM_026402)	(NM_134394)	(XM_005601995)	(XM_003132682)	(NM_001075364)
**Chicken Atg5**	80.11	75.55	72.09	68.96	68.84	79.53
(NM_001006409)	(NM_004849)	(NM_053069)	(NM_001014250)	(XM_005596852)	(NM_001037152)	(NM_001034579)
**Chicken Atg9A**	71.97	71.37	66.47	74.7	80.01	73.46
(NM_001034821)	(BC_065534)	(NM_001288612)	(NM_001014218)	(XM_001493040)	(NM_001190275)	(NM_001034706)
**Chicken Atg10**	59.76	64.64	65.6	62.35	69.42	59.12
(XM_424902)	(NM_001131028)	(NM_025770)	(NM_001109505)	(XM_005599592)	(NM_001190281)	(NM_001083531)
**Chicken Atg12**	65.87	52.69	54.48	75.89	61.67	66.99
(XM_003643073)	(NM_004707)	(NM_026217)	(NM_001038495)	(XM_003362836)	(NM_001190282)	(NM_001076982)
**Chicken Atg14**	65.13	64.74	65.33	74.53	65.34	64.05
(XM_426476)	(NM_014924)	(NM_172599)	(NM_001107258)	(XM_001914860)	(XM_001924990)	(NM_001192099)
**Chicken Atg13**	65.84	67.14	75.47	69.47	61.84	63.58
(XM_003641387)	(NM_001205119)	(NM_145528)	(NM_001271212)	(NM_001242529)	(XM_003122826)	(NM_001076812)
**Chicken Atg7**	75.77	71.52	76.28	74.05	76.15	69.17
(NM_001030592)	(NM_006395)	(NM_001253717)	(NM_001012097)	(XM_005600372)	(NM_001190285)	(NM_001142967)
**Chicken Atg4B**	79.19	78.26	50.76	78	75.8	76.06
(NM_213573)	(NM_013325)	(NM_174874)	(NM_001025711)	(XM_005610806)	(NM_001190283)	(NM_001001170)
**Chicken Atg4A**	69.32	66.82	65.76	73.15	72.51	78.03
(NM_001271986)	(NM_052936)	(NM_174875)	(NM_001126298)	(XM_005614404)	(XM_005657911)	(NM_001001171)
**Chicken Atg16L1**	67.77	68.31	70.67	65.24	78.4	67.59
(XM_003641751)	(NM_030803)	(NM_001205391)	(NM_001108809)	(XM_005610723)	(NM_001190272)	(NM_001191389)
**Chicken UVRAG**	77.23	76.16	73.96	76.6	-	75.73
(NM_001030839)	(AB.12958)	(NM_178635)	(NM_001107536)	(XM_001917231)		(NM_001193026)
**Chicken AMBRA1**	70.69	73.38	73.4	77.09	74.5	72.66
(XM_001233288)	(NM_001267782)	(NM_172669)	(NM_001134341)	(XM_005598075)	(XM_003122844)	(NM_001034522)

Genbank accession number is indicated for each gene and each species between brackets.

### Statistical analyses

Data were analysed by two-factor ANOVA with tissue and gender (for chicken) and tissue and genotype (for quail) as classification variables. If ANOVA revealed significant effects, the means were compared by Tukey multiple range test using the Graph Pad Prism version 6.00 for Windows, Graph Pad Software, La Jolla California USA. Differences were considered significant at *P*<0.05.

## Results

### Tissue distribution of autophagy-related genes in chickens and quails

**Figure 1:**
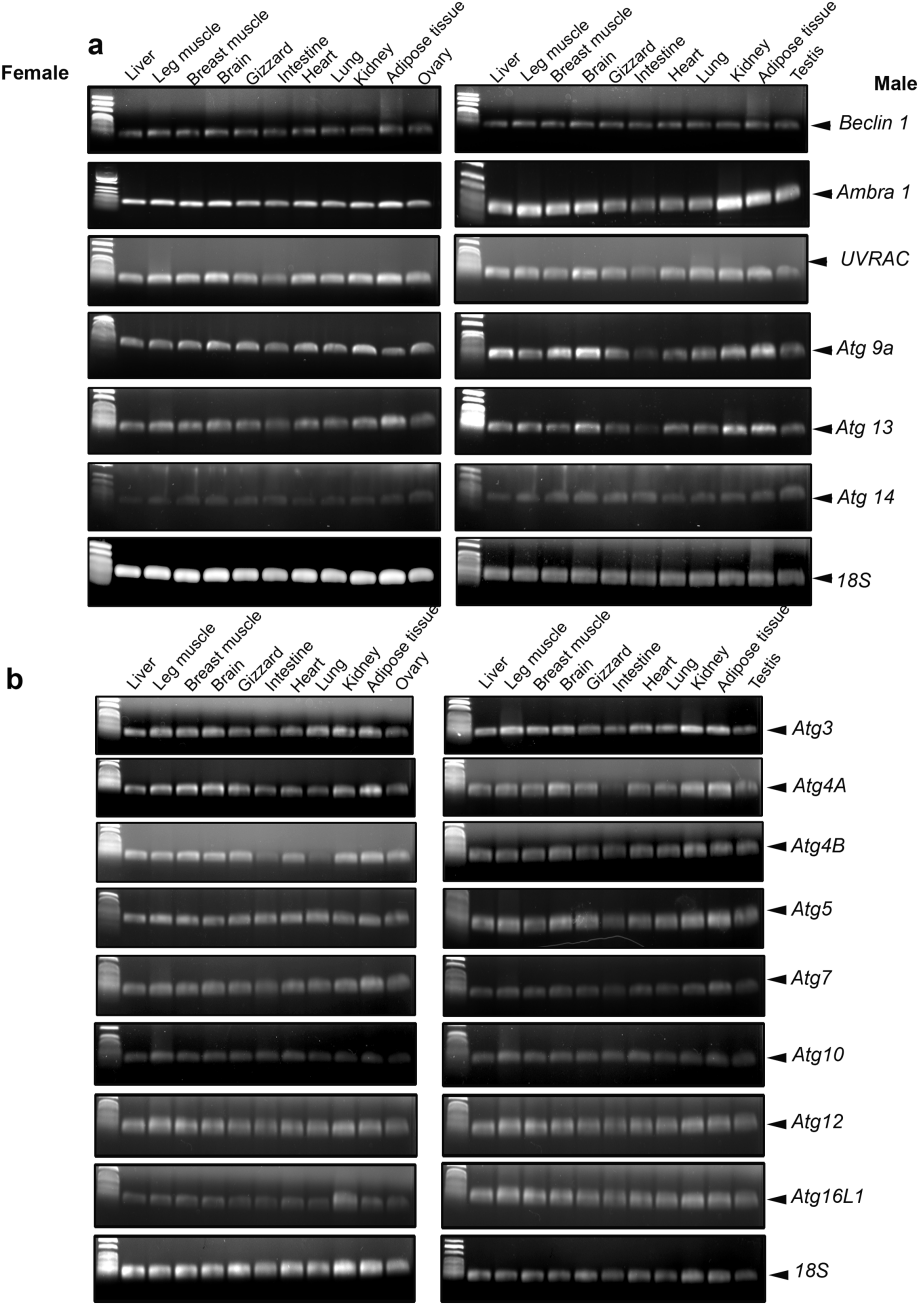
Characterization of autophagosome initiation- (a) and elongation-related genes (b) in various tissues of male and female Red Jungle Fowl (*Gallus gallus*) by RT-qPCR as described in materials and methods. Signals were visualized by agarose gel electrophoresis.

**Figure 2:**
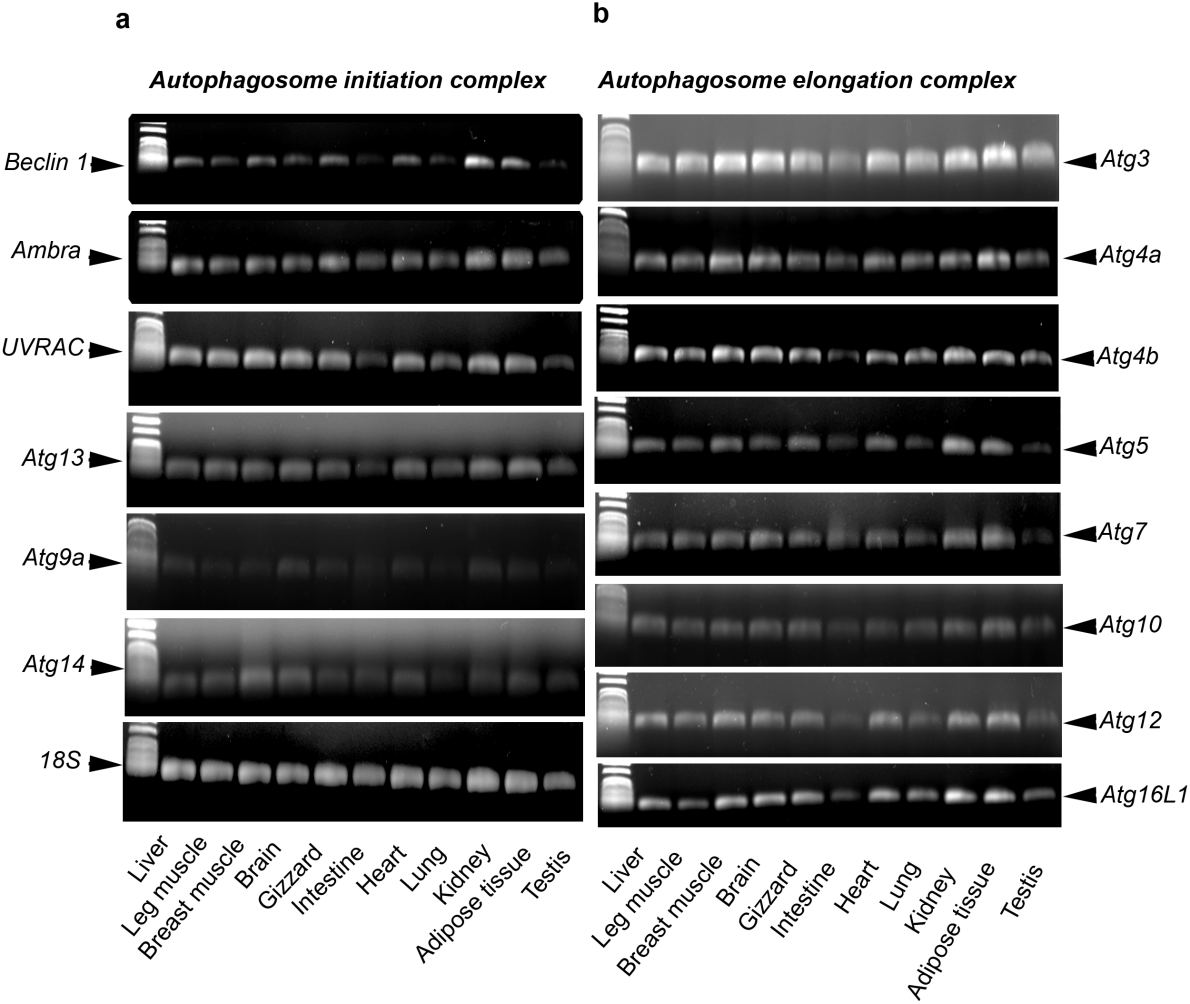
Characterization of autophagosome initiation- (a) and elongation-related genes (b) in various tissues of stress-sensitive (S) and stress-resistant (R) male Japonica quail (*Coturnix coturnix Japonica*) using RT-qPCR. Signals were visualized by agarose gel electrophoresis.

Since the role of autophagy-related genes is still unknown in avian species, we classified them in the following sections based on their roles in yeast and mammals. Gene complexes involved in autophagosome initiation (beclin1, Ambra1, UVRAG, Atg9a, Atg13 and Atg14) and elongation (Atg3, Atg4A, Atg4B, Atg5, Atg7, Atg10, Atg12 and Atg16L1) were ubiquitously expressed in chicken and quail. Only one band of the predicted size for each gene was observed in liver, leg and breast muscle, brain, gizzard, intestine, heart, lung, kidney, adipose tissue, ovary and testis (Fig. 1a, b and Fig. 2a, b). The sequences of the fragments were identical (100%) to these previously described in GenBank (for accession number, see Table 1 and 2).

### Expression of autophagosome initiation complex in different tissues of female and male chickens

**Figure 3:**
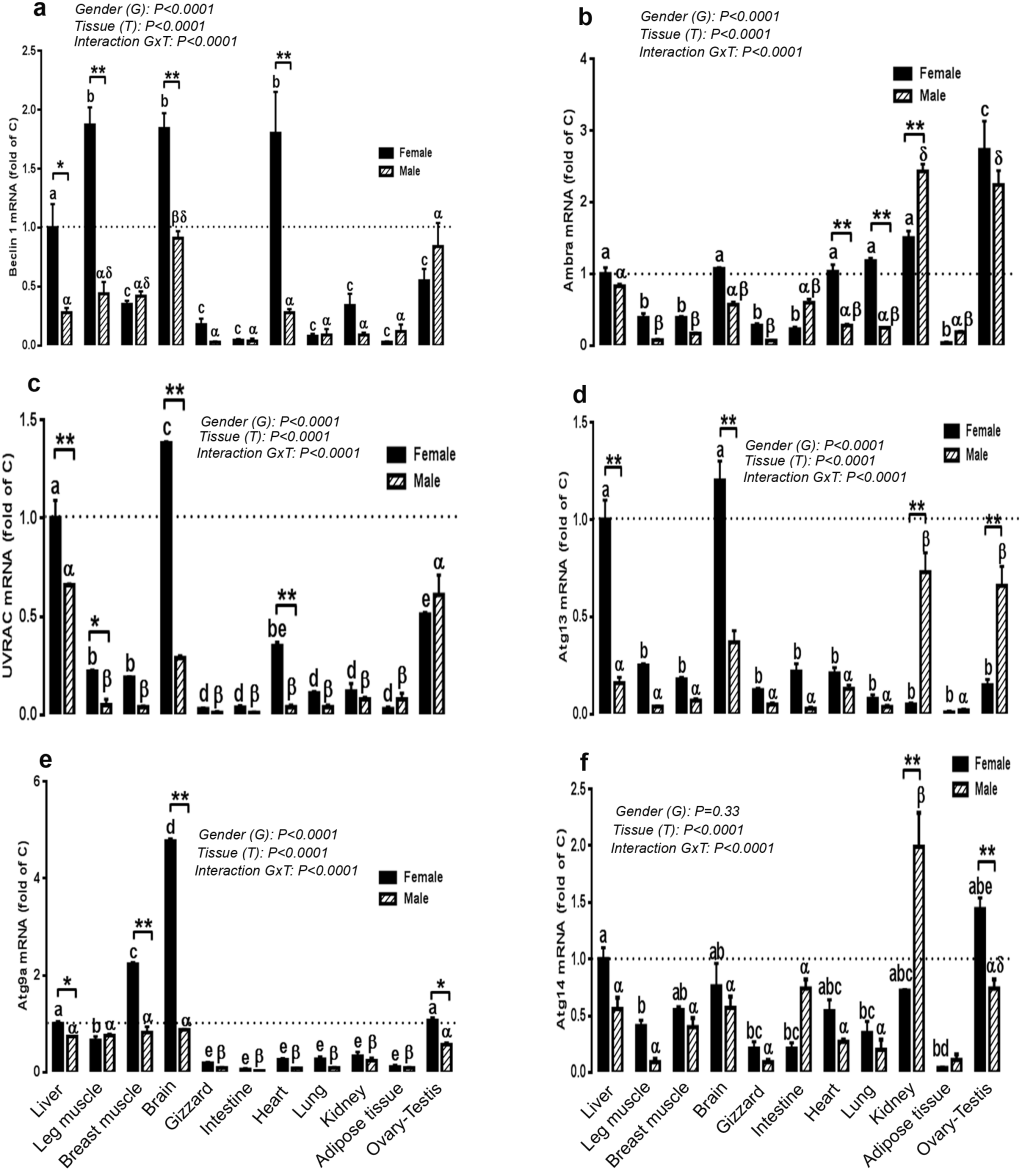
Comparison of relative expression of autophagosome initiation-related genes in various tissues of male and female Red Jungle Fowl. Total RNA from each tissue was DNAse-treated, reverse transcribed, and subjected to real-time quantitative PCR as described in material and methods. Samples were run in duplicate, and the average threshold cycle (Ct) values were determined for the target and houskeeping genes. Relative quantity of autophagy genes was determined by the 2^−ΔΔCt^ method [58]. Data are presented as mean ± SEM (n = 6 for each gender and each tissue). * Sex-matched differences among tissues (*P<0.05 and **P<0.01). Different letters indicate tissue-matched differences among gender (a–e, difference between tissues within female and α-δ indicate differences between male tissues).

The autophagosome initiation complex was expressed in all tissues examined in female and male Red Jungle fowl chickens. In female chickens, beclin 1 was highly expressed in the heart, brain and leg muscle, followed by the ovary (Fig. 3a). Ambra 1 mRNA abundance was higher in the ovary followed by kidney, lung, heart, brain and the liver (Fig. 3b). UVRAG and Atg13 genes were highly expressed in the brain and the liver (Fig. 3c and d). Atg9a mRNA levels were greater in the brain followed by breast muscle, ovary and liver (Fig. 3e). The highest amount of Atg14 mRNA was found in the ovary followed by the liver, brain, kidney, heart and the breast muscle (Fig. 3f). In males, however, the highest levels of beclin1 mRNA were observed in brain and testis, followed by leg and breast muscle and liver (Fig. 3a). The greatest expression of Ambra 1 and Atg13 genes was found in kidney and testis (Fig. 3b). UVRAG gene was highly expressed in liver and testis followed by the brain (Fig. 3c). Atg9a mRNA abundance was high in the liver, brain, leg and breast muscle followed by the testis (Fig. 3e). Atg14 gene expression was high in kidney followed by testis, intestine, brain, liver and breast muscle (Fig. 3f).

Interestingly, when tissues from the two genders were plotted together, female chickens exhibited greater hepatic abundance of beclin 1 (3.57 fold, *P*<0.05), UVRAG (1.5 fold, *P*<0.01), Atg13 (6.25 fold, *P*<0.01), and Atg9a mRNA (1.35 fold, *P*<0.05) compared to males (Fig. 3a, c, d and e). Females also exhibited significant higher expression of the following genes: beclin1 in leg muscle (4.25 fold), brain (2 fold) and heart (6.24 fold, Fig. 3a), Ambra 1in lung and heart (3.67 and 4.72 fold respectively, Fig. 3b), UVRAG in leg muscle, brain, and heart (4.4, 4.75, and 8.75 fold respectively, Fig. 3c), Atg13 in brain (3.24 fold, Fig. 3d), Atg9a in breast muscle, brain and ovary (2.71, 5.42, and 1.87 fold respectively, Fig. 3e) and Atg14 in ovary (1.94 fold, Fig. 3f) compared to males. However, in male chickens, Ambra 1 and Atg14 gene expression was higher in kidney (1.62 and 2.76 fold respectively, *P*<0.01, Fig. 3d and f) and Atg13 mRNA levels were higher in testis and kidney (4.4 and 14.6 fold respectively, *P*<0.01, Fig. 3d) compared to female.

### Expression of autophagosome elongation complex in different tissues of female and male chickens

**Figure 4:**
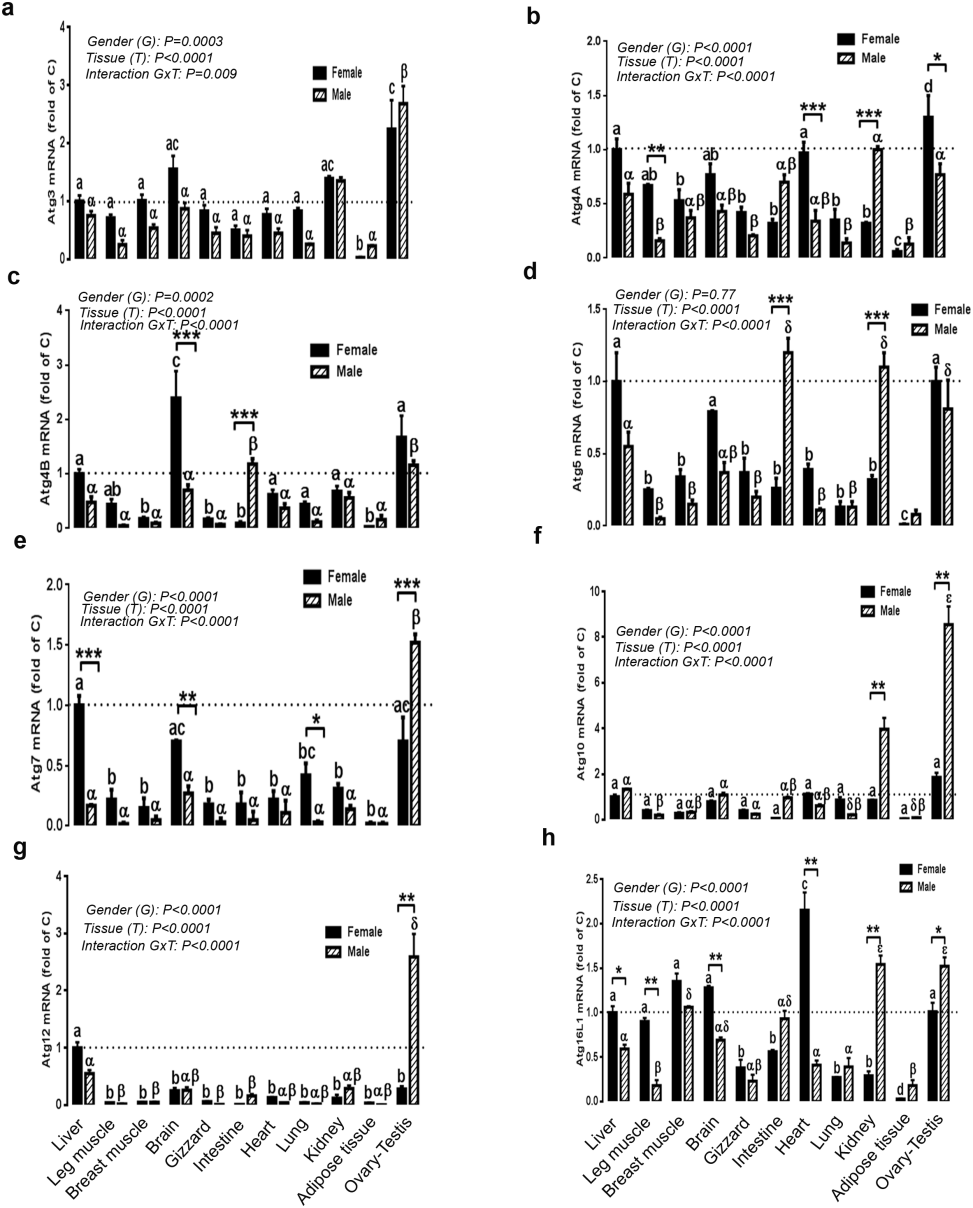
Comparison of relative expression of autophagosome elongation-related genes in various tissues of male and female Red Jungle Fowl. Total RNA from each tissue was DNAse-treated, reverse transcribed, and subjected to real-time quantitative PCR as described in material and methods. Sample were run in duplicate, and the average threshold cycle (Ct) values were determined for the target and houskeeping genes. Relative quantity of autophagy genes was determined by the 2^−ΔΔCt^ method [58]. Data are presented as mean ± SEM (n = 6 for each gender and each tissue). * Sex-matched differences among tissues (*P<0.05 and **P<0.01). Different letters indicate tissue-matched differences among gender (a–e, difference between tissues within female and α-δ indicate differences between male tissues).

As the initiation complex, the autophagosome elongation complex is ubiquitously expressed in both male and female chickens. In females, the highest amount of Atg3 mRNA was found in the ovary followed by brain and kidney. Atg4A mRNA was abundant in the liver, heart, brain, leg and breast muscle. Atg5 gene expression was higher in the liver, brain, gizzard, heart and breast muscle (Fig. 4a, b and d). Atg4B gene was highly expressed in brain, followed by ovary and liver (Fig. 4c). Atg7 and Atg12 mRNA were abundant in liver followed by ovary, brain, lung, kidney and leg muscle for Atg7 and ovary and brain for Atg12 (Fig. 4e and g). Atg16L1 mRNA levels were high in heart followed by breast muscle, brain, liver, ovary and leg muscle (Fig. 4h). In males, however, the highest amount of Atg3, Atg4B, Atg7, Atg10, Atg12 and Atg16L1 mRNA was found in testis followed by kidney, brain and liver for Atg3, intestine, brain, liver and kidney for Atg4B, brain, liver and kidney for Atg7, kidney, liver, brain, intestine and heart for Atg10, liver, brain and kidney for Atg12, and kidney, breast muscle, intestine, brain and liver for Atg16L1 (Fig. 4a, c, e, f, g and h). Atg4A and Atg5 mRNA levels were high in kidney and intestine followed by testis, liver, brain and breast muscle for Atg4A and testis, liver and brain for Atg5 (Fig. 4b and d). Importantly, when we profile the autophagosome elongation complex for each tissue within the two genders, only a few genes showed gender- and tissue-dependent pattern. Female chickens displayed significant high expression of Atg4A in leg muscle, heart and ovary (4.18, 2.85 and 1.68 fold, respectively, Fig. 4b), Atg4B in the brain (3.41 fold, Fig. 4c), Atg7 in liver, brain and lung (5.88, 2.59 and 14 fold, respectively, Fig. 4e), and Atg16L1 in liver, leg muscle, brain and heart (1.69, 5, 1.85 and 5.24 fold, respectively, Fig. 4h) compared to males. However, male chickens exhibited significant higher levels of Atg4A mRNA in kidney (3.12 fold, Fig. 4b), Atg4B mRNA in intestine (13.11 fold, Fig. 4c), Atg5 mRNA in kidney and intestine (3.43 and 4.61 fold, respectively, Fig. 4d), Atg7 mRNA in testis (2.17 fold, Fig. 4e), Atg10 in testis and kidney (4.61 and 4.65 fold, respectively, Fig. 4f), Atg12 in testis (9.25 fold, Fig. 4g), and Atg16L1 in testis and kidney (1.52 and 5.31 fold, Fig. 4h). Atg3 gene expression did not differ between male and female in every studied tissue (Fig. 4a).

### Expression of autophagosome initiation complex in different tissues of S and R quail lines

**Figure 5:**
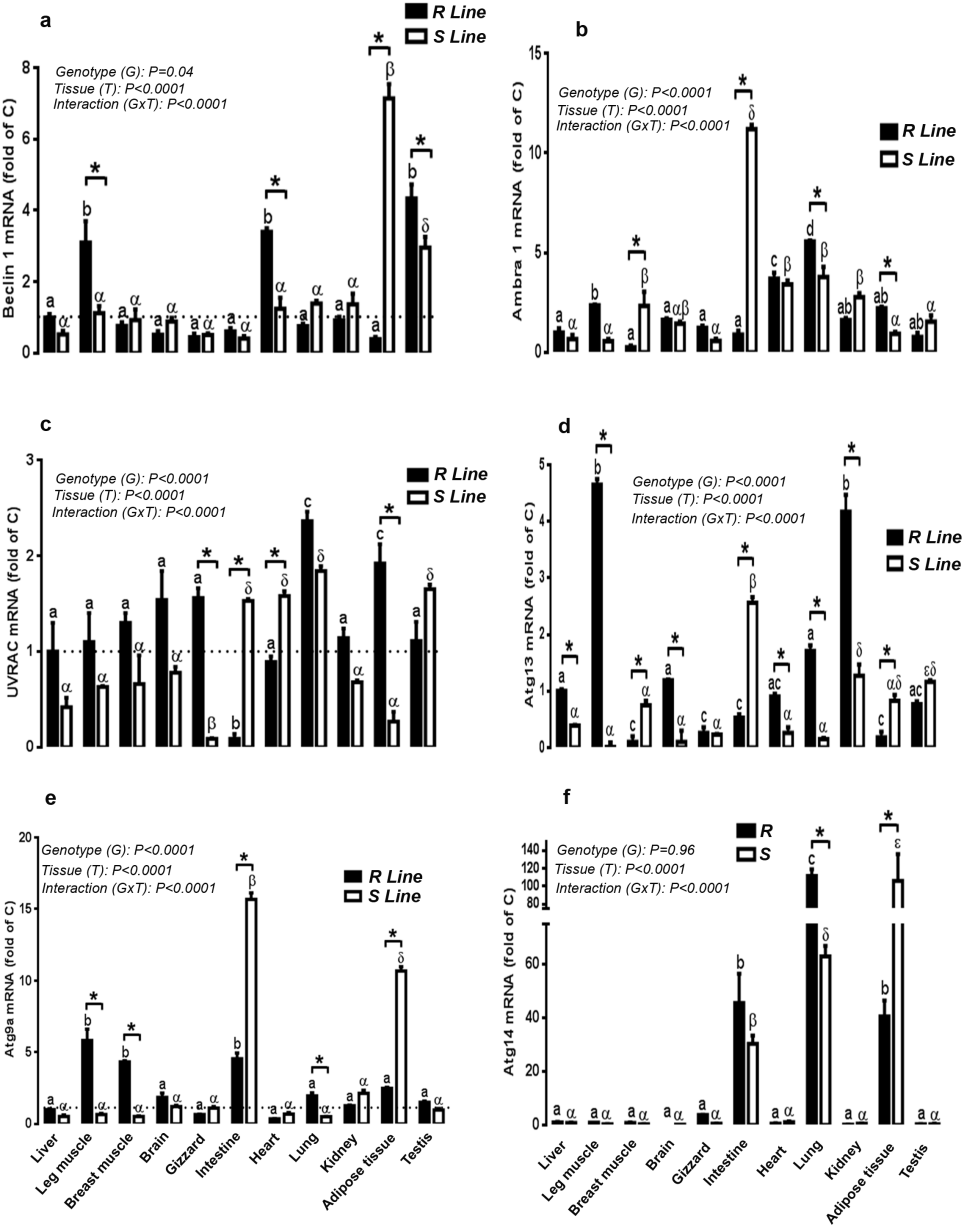
Relative expression of autophagosome initiation-related genes in various tissues of R ans S male Japonica quail lines. Total RNA from each tissue was DNAse-treated, reverse transcribed, and subjected to real-time quantitative PCR. Sample were run in duplicate, and the average threshold cycle (Ct) values were determined for the target and houskeeping genes. Relative quantity of autophagy genes was determined by the 2^−ΔΔCt^ method [58]. Data are presented as mean ± SEM (n = 6 for each line and each tissue). * Line-matched differences among tissues (*P<0.05). Different letters indicate tissue-matched differences among Lines (a–c, difference between tissues within R line and α-ε indicate differences between tissues within S line).

The autophagosome initiation complex was expressed in all tissues examined in quail. Beclin 1 mRNA levels were abundant in testis, heart and leg muscle of R line and in adipose tissue and testis of S line (Fig. 5a). The highest amount of Ambra1 was found in lung, heart and leg muscle of R line and in intestine, lung, heart, kidney, and breast muscle of S line (Fig. 5b). The UVRAG expression was high in lung and adipose tissue of R quail and in lung followed by testis, lung, heart and intestine in S line (Fig. 5c). The highest amount of Atg13 was found in leg muscle and kidney of R line and in intestine followed by kidney and testis in S line (Fig. 5d). Atg9a gene was highly expressed in leg and breast muscle followed by intestine in R line and in intestine and adipose tissue in S line (Fig. 5e). Atg14 gene expression was found to be high in lung, adipose tissue and intestine in both lines (Fig. 5f). When tissues from the two lines were plotted together, R line exhibited significant higher mRNA abundance of beclin 1 in leg muscle, heart, and testis (2.76, 2.72 and 1.46 fold, respectively), Ambra 1 in lung and adipose tissue (1.46 and 2.37 fold, respectively), UVRAG in gizzard and adipose tissue (17.3 and 7.1 fold, respectively), Atg13 in liver, leg muscle, brain, heart, lung and kidney (2.56, 46.5, 11.9, 3.4, 11.4 and 3.2 fold, respectively), Atg9a in lung, leg and breast muscle (3.86, 8.8 and 8.3 fold, respectively), and Atg14 in in lung (1.77 fold) compared to S line (Fig. 5a, b, c, d, e and f). However, S line exhibited significant higher levels of beclin 1 in adipose tissue (17.8 fold), Ambra 1 in breast muscle and intestine (9 and 12.5 fold, respectively), UVRAG in intestine and heart (17 and 1.7 fold, respectively), Atg13 in breast muscle, intestine and adipose tissue (7.5, 4.8 and 4.6 fold, respectively), Atg9a in intestine and adipose tissue (3.4 and 4.2 fold, respectively), and Atg14 in adipose tissue (2.6 fold) compared to R line (Fig. 5a, b, c, d, e and f).

### Expression of autophagosome elongation complex in different tissues of R and S quail lines

**Figure 6:**
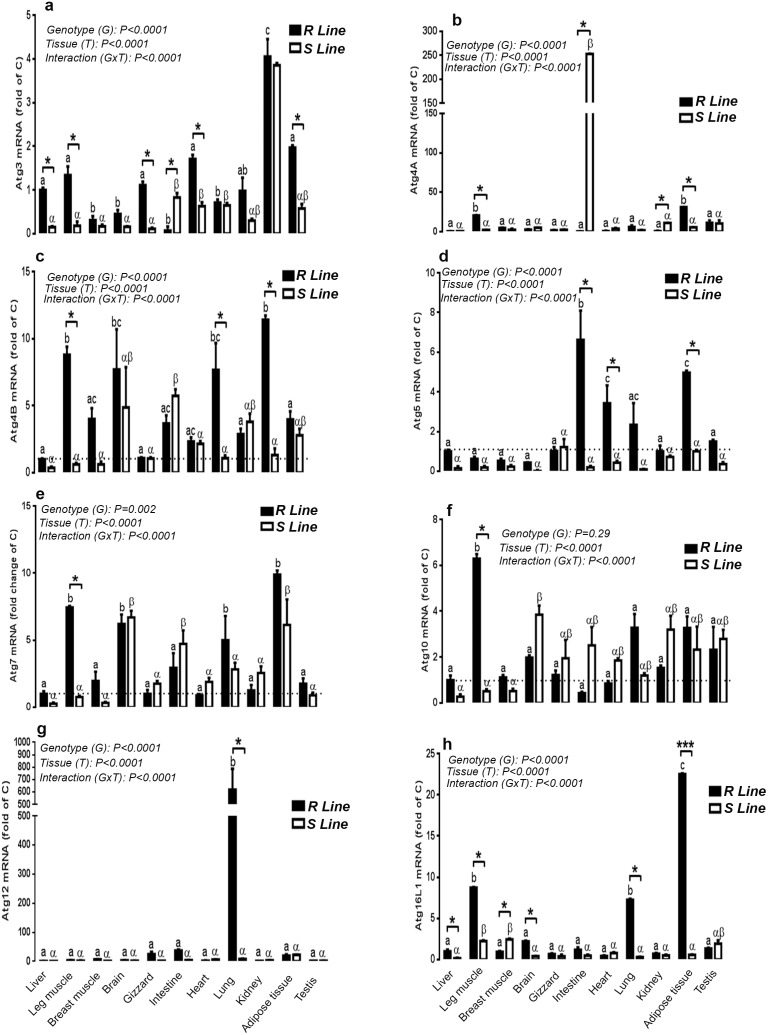
Comparison of relative expression of autophagosome elongation-related genes in various tissues of R and S male Japonica quail lines. Total RNA from each tissue was DNAse-treated, reverse transcribed, and subjected to real-time quantitative PCR. Sample were run in duplicate, and the average threshold cycle (Ct) values were determined for the target and houskeeping genes. Relative quantity of autophagy genes was determined by the 2^−ΔΔCt^ method [58]. Data are presented as mean ± SEM (n = 6 for each line and each tissue). * Genotype-matched differences among tissues (*P<0.05 and ***P<0.001). Different letters indicate tissue-matched differences among genotype (a, b, difference between tissues within R line and α-β indicate differences between tissues within S line). 28.

Atg3 gene was highly expressed in adipose tissue of both lines followed by testis, heart, leg muscle, gizzard and liver in R line and by testis, lung and heart in S line (Fig. 6a). The highest amount of Atg4a mRNA was found in leg muscle and adipose tissue of R line and in intestine of S line (Fig. 6b). Atg4b mRNA levels, however, was high in adipose tissue, leg muscle, brain and lung of R line and in intestine and brain of S line (Fig. 6c). Atg5 was highly expressed in intestine, heart, adipose tissue and lung of R line and its expression remain unchanged between the examined tissues of S line (Fig. 6d). Atg7 mRNA abundance was found to be high in adipose tissue, leg muscle, brain and lung of R line and in brain, adipose tissue and intestine of S line (Fig. 6e). Atg10 was highly expressed in leg muscle in R line and in brain of S line (Fig. 6f). The highest amount of Atg12 mRNA was found in lung of R line but did not differ between tissues in S line (Fig. 6g). Atg16L1 gene expression was high in adipose tissue followed by leg muscle and lung in R line and in leg and breast muscle of S line (Fig. 6h). Interestingly, when the two genotypes are plotted together, R line displayed significant high levels of Atg3 in liver, leg muscle, gizzard, heart, and testis (6.6, 7.4, 10, 2.7, and 3.3 fold, respectively), Atg4a in leg muscle and adipose tissue (9.4 and 5.6 fold, respectively), Atg4b in leg muscle, lung and adipose tissue (13.9, 7, and 8.7 fold, respectively), Atg5 in intestine, heart and adipose tissue (33, 8, and 5 fold, respectively), Atg7 and Atg10 in leg muscle (9.7 and 12.3 fold, respectively), Atg12 in lung (77 fold), and Atg16L1 in liver, leg muscle, brain, lung, and adipose tissue (6.25, 3.89, 5.2, 25, and 39 fold, respectively) compared to S line (Fig. 6a–h). However S line exhibited higher mRNA levels of Atg3 in intestine (4.8 fold), Atg4a in kidney (8.4 fold), and Atg16L1 in breast muscle (2.6 fold) (Fig. 6a–h).

### Alignment and phylogenetic tree analysis of chicken autophagy-related genes with other sources

**Figure 7:**
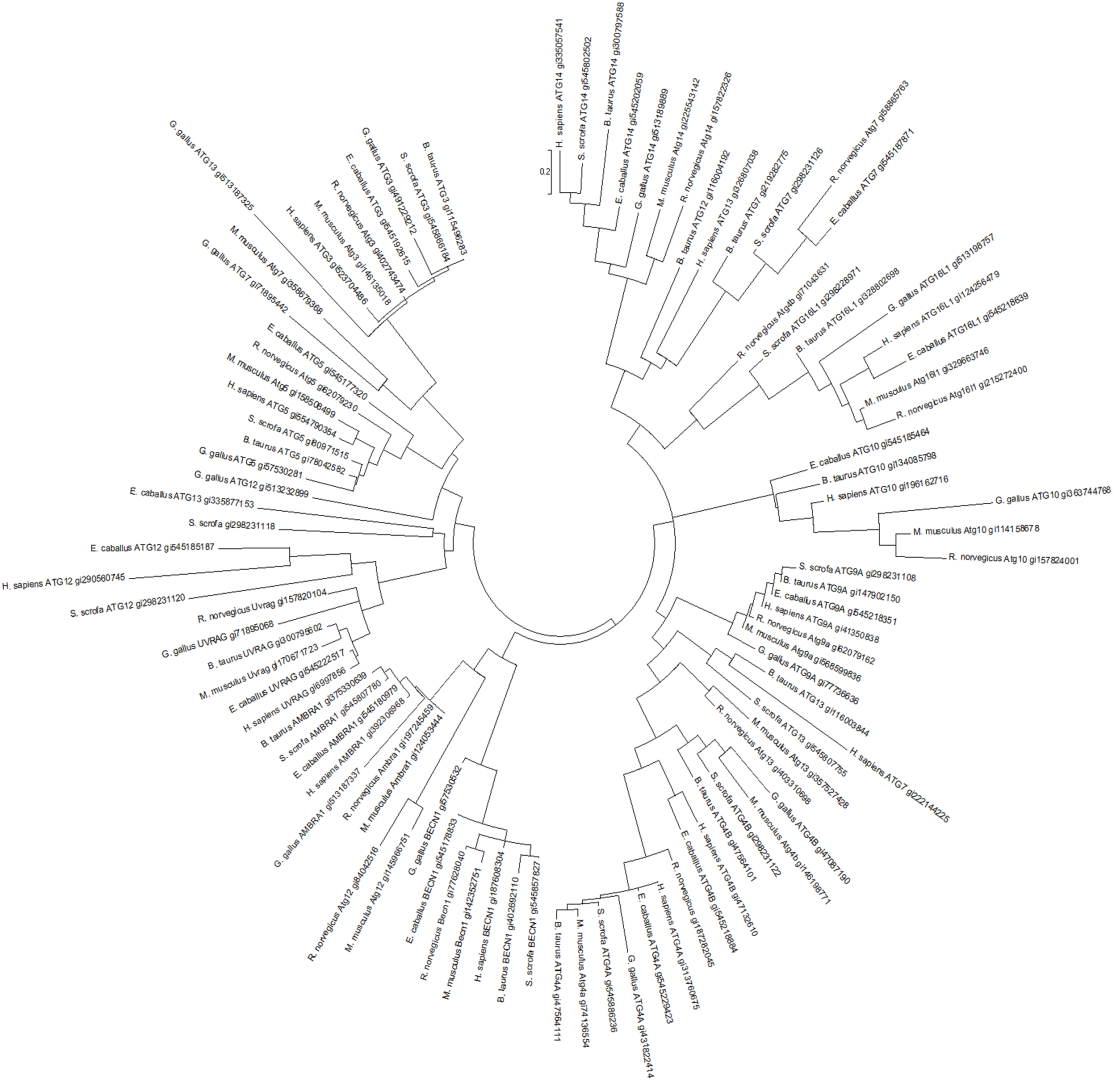
Phylogenetic relationships among chicken autophagy-related genes and their mammalian orthologs were inferred using the neighbor-joining method in MUSCLE alignment and MEGA6. Scale bar indicates the substitution rate per residue. Genbank accession numbers are included in the phenogram and in Table 2.

Comparison of the nucleotide sequences of autophagosome initiation and elongation-related genes between chickens and other species showed low to high similarity (52.6%–91.5%) (Table 2). Phylogenetic analysis indicates that chicken Atg4b, Atg7, Atg9, Atg10, Atg14, Atg16L1, and Ambra1 are more closely related to the mouse orthologs however Atg3 and Atg4a are closely related to the pig orthologs, beclin1 is closely related to the horse ortholog, UVRAG is closely related to the rat ortholog and Atg5 is closely related to the bovine ortholog (Fig. 7).

## Discussion

Autophagy is an evolutionary conserved catabolic process regulating the degradation of a cell’s own components through the lysosomal machinery [38]. It plays a key homeostatic role in every cell type to preserve the balance between the synthesis, degradation, and subsequent recycling of cellular components [39]. Currently, more than thirty different autophagy-related genes have been identified by genetic screening in yeast, and many of these genes are conserved in plants, flies and mammals [40]. However, data in birds are scarce. Here, we report for the first time the characterization of fourteen avian genes involved in the autophagosome initiation and elongation. All genes had high basal expression levels in every examined tissue from chicken and quail maintained under normal (low stress) physiological conditions. These data indicate that avian cells are also equipped with the autophagy system which may be involved in numerous vital cell processes including cellular homeostasis, tissue development and a defense mechanism against aggregated proteins, damaged organelles and infectious agents.

The MUSCLE alignment and the phenogram construction of nucleotide sequences of chicken autophagy-related genes and their mammalian orthologs show high homology and indicate that the evolutionary relationship is consistent with the consensus view of vertebrate evolution. Although the evolutionary conservation of the autophagy pathway, many of the mechanistic breakthroughs in delineating how autophagy is regulated and executed at the molecular level have been made in yeast [41]. Here, our quantitative real-time PCR analysis revealed that the expression of avian autophagy-related genes is tissue specific suggesting a tissue-dependent modulation mechanism of autophagy under physiological conditions and corroborating results from previous studies in rats [42] and mice [43]. This may reflect fundamental differences in the fate of the tissue and/or cells, the role of the autophagy-related genes and their interactions in rapidly dividing versus post-mitotic cells. For instance, inhibition of autophagy using Atg7 small interfering RNA inhibited cell death during starvation in neuronal cells, but increased cell death in fibroblasts [42]. Most likely there is no single autophagy pathway across all tissues, and even within the same tissue, multiple effectors and mediators may exist.

We have also demonstrated that avian autophagy-related gene expression is gender dependent. Although the underlying mechanism(s) for this apparent sexual dimorphic expression is (are) unknown, the results are not surprising because sex-dependent differences in the activation of the autophagic cytoprotection pathway have been reported in mammals [42], [44], [45]. The gender-associated differences in autophagy-related genes observed in our study could be a result of physiological, morphological, and hormonal differences between both sexes. Indeed, variations in androgen levels have been shown to be an important factor for the development of autophagy [44]. Sobolewska and co-workers showed that 17 beta-estradiol and progesterone exerted stimulatory effects on autophagy in bovine mammary epithelial cells [46]. Furthermore, a sexual dimorphism of estrogen receptor (ERα and ERβ) expression was observed in basal vascular smooth muscle cells (VSMC) and their regulation by oxidative stress was also found to be gender-dependent (alteration in female but not in male VSMC) [47]. Additionally, The downstream cascades mediating the cardio-protective effects of ERβ in tumor necrosis factor receptor-2 (TNFR2) knockout mice has been shown to be gender-dependent with activation and translocation of signal transducer and activator of transcription 3 (STAT3) in female and decrease of c-jun N-terminal kinase (JNK) in male [48]. Because STAT3 activation has been associated with autophagy processes [49], and JNK phosphorylates B-cell lymphoma 2 (Bcl-2) triggering its release from beclin 1 in response to various stimuli [50], the existence of sexual dimorphic autophagy signaling cascades is very likely. As autophagy is tightly linked to starvation and fatty acid metabolism [42], it is possible that other sex-dependent hormones known to be involved in the regulation of energy homeostasis and lipid metabolism such as leptin, insulin and ghrelin, may affect autophagy-related gene expression as previously reported in mammals [51]–[54].

Interestingly, we also found that the expression of autophagy-related genes is tissue- and genotype-dependent in male Japanese quails. These quail lines were divergently selected for circulating corticosterone response to restraint stress [33]. The high stress or sensitive line had, in general, high plasma corticosterone levels, high mortality, increased bacterial colonization, high fearfulness, low sexual activity and high stress-induced osteoporosis compared to their low stress (resistant) counterpart [33], [55]–[58]. Although the role and the regulation of the autophagy-related genes are still unknown in avian species, the high expression of most studied genes in the R line suggest that the autophagy might be a protective mechanism in response to stress and might be involved in the aforementioned behavior and physiological differences between the two quail lines. For instance, inhibition of autophagy has been shown to aggravate the effect of glucocorticoid on cell viability of chicken primary osteocytes [59] which may explain the high stress-induced corticosterone levels and osteoporosis in S lines. Furthermore, sperm quality has been linked to autophagy (LC3B) processing [60] which may support the heightened reproductive efficiency of male R quails which are characterized by increased testis expression of beclin1 and Atg3 genes. Since autophagy has been, recently, reported to be associated with the development of learning and memory in fear conditioning [61], the low expression of Atg13 and Atg16L1 in the brain of S line might be involved in their high fearfulness. Intriguingly, the expression of autophagosome initiation (Ambra 1, UVRAG, Atg13, Atg9a) and elongation genes (Atg3 and Atg4a), except Atg5, was higher in the intestine of S line compared to the R line. The biological significance of this differential expression is not known at this time and further studies are warranted.

In conclusion, the characterization herein of several genes involved in autophagosome initiation and elongation will open new research avenues to understand the regulation and the roles of autophagy in avian species maintained under physiological and pathophysiological conditions. Further studies are warranted to identify and characterize genes involved in autophagosome maturation in birds. The present study also provides proof of principle evidence supporting gender- and genotype-dependent differences in autophagy in avian species and better insight into the underlying mechanisms may ultimately help to develop new management tools for poultry production improvement. The quail lines may be a useful model to study stress-related disorder in human and develop therapeutic strategies.
